# Direct Umbilical Vein Injection of Epinephrine with Cut-Cord Milking in an Ovine Model of Neonatal Resuscitation

**DOI:** 10.3390/children11050527

**Published:** 2024-04-28

**Authors:** Payam Vali, Peggy Chen, Evan Giusto, Amy Lesneski, Morgan E. Hardie, Heather K. Knych, Deepika Sankaran, Ziad Alhassen, Houssam M. Joudi, Satyan Lakshminrusimha

**Affiliations:** 1 Division of Neonatology, Department of Pediatrics, University of California Davis, Sacramento, CA 95817, USA; pvali@ucdavis.edu (P.V.); dsankaran@ucdavis.edu (D.S.); hmjoudi@ucdavis.edu (H.M.J.); 2 Division of Neonatology, Miller Children’s & Women’s Hospital Long Beach, Long Beach, CA 90806, USA; peggy.chen@pediatrix.com; 3 D-5 Neonatal Units, Patient Care Services, University of California Davis, Sacramento, CA 95817, USA; 4 Department of Stem Cell Research, University of California Davis, Sacramento, CA 95817, USA; 5 School of Veterinary Medicine, University of California Davis, Davis, CA 95616, USA; 6 K.L. Maddy Equine Analytical Pharmacology Laboratory, Department of Veterinary Molecular Biosciences, School of Veterinary Medicine, University of California Davis, Davis, CA 95616, USA; 7 Division of Neonatology, Children’s Hospital of Orange County, Orange, CA 92868, USA

**Keywords:** delivery room, chest compressions, epinephrine, umbilical cord milking

## Abstract

Background: An umbilical venous catheter (UVC) is the preferred route of epinephrine administration during neonatal resuscitation but requires specialized equipment, expertise, and time. Hypothesis: Direct injection of epinephrine into the umbilical vein (UV) followed by milking a ~20 cm segment of cut umbilical cord to flush the epinephrine (DUV + UCM) will lead to a quicker administration and earlier return of spontaneous circulation (ROSC) compared with epinephrine given through a UVC. Design: Eighteen near-term asphyxiated lambs were randomized to receive a low-UVC or DUV + UCM of epinephrine at 0.02 or 0.03 mg/kg doses. Outcome measures: A total of 16/18 lambs achieved ROSC with a similar mean (±SEM) time to ROSC [DUV + UCM vs. low-UVC (4.67 ± 0.67 vs. 3.99 ± 0.58 min); *p* = 0.46]. Two out of ten lambs in the DUV + UCM group required UVC placement for additional epinephrine. The administration of the first dose of epinephrine was similar (DUV + UCM—2.97 ± 0.48 vs. UVC—4.23 ± 0.58 min; *p* = 0.12). Both methods yielded similar epinephrine concentrations (peak concentrations of 253 ± 63 and 328 ± 80 ng/mL for DUV + UCM and UVC EPI, respectively). Conclusions: DUV + UCM resulted in a ROSC success of 78% following the first epinephrine dose and showed similar epinephrine concentrations to UVC. Clinical studies evaluating DUV + UCM as an alternate route for epinephrine while intravenous access is being established are warranted.

## 1. Introduction

Globally, perinatal asphyxia accounts for a quarter of the approximate 2.5 million neonatal deaths every year [1]. Neonates who require chest compressions and epinephrine during resuscitation in the delivery room (DR) have high morbidity and mortality [2,3,4]. A quicker time to the first dose of epinephrine has been shown to increase the odds of return of spontaneous circulation (ROSC) in the delivery room (DR) [5] and may improve survival and outcomes [6]. Epinephrine is the only medication currently recommended for neonatal resuscitation by the International Liaison Committee on Resuscitation (ILCOR) [7,8]. For severely bradycardic (heart rate [HR] < 60 beats per minute [bpm]) or asystolic newborns receiving effective ventilation and chest compressions, the American Academy of Pediatrics Neonatal Resuscitation Program (NRP) recommends administering epinephrine, preferably by the intravenous (IV) or, alternatively, the intraosseous (IO) route [8,9]. Another option is the administration of epinephrine through the endotracheal tube (ETT) while IV access is being established [10,11].

For the administration of epinephrine, the NRP recommends the placement of a low-lying umbilical venous catheter (UVC) or an intraosseous needle. UVC placement requires time, advanced skills, and specialized materials. There is significant variation in the availability of equipment and neonatal resuscitation skills among clinicians who work in DR settings [12]. Simulations and clinical studies have shown that the median time to administer epinephrine ranges from five to seven minutes, even when resuscitation is performed at a large referral hospital with trained, experienced neonatal staff [3,13,14]. Epinephrine administered by the ETT is quicker but has been shown to be less effective compared with the IV route [3,10,15,16]. In a retrospective study among asystolic neonates in the DR, only 3/37 (8%) achieved ROSC with ETT epinephrine alone [17,18]. Umbilical cord milking in non-vigorous-term infants is associated with a lower incidence of hypoxic–ischemic encephalopathy (HIE) and a reduced need for respiratory support [19,20,21]. Cut-umbilical cord milking is an alternate approach to placental transfusion. Cut-umbilical cord milking is an approach to cord management that involves cutting a long segment of the umbilical cord attached to the neonate and milking it towards the newly born infant. It has been studied in clinical trials. However, it has not been evaluated in conjunction with umbilical vein injection of medications such as epinephrine in a model of cardiac arrest [22,23].

The purpose of this study was to compare the current recommended practice of epinephrine administration through a low-lying UVC followed by a saline flush (UVC EPI) with an initial dose via a direct umbilical vein injection followed by the milking of a 20 cm length of cut umbilical cord (DUV + UCM). Our hypothesis was that administration of IV epinephrine directly into the umbilical vein (UV) at the base of the umbilicus followed by cord milking would be quicker and achieve early but similar rates of ROSC compared with UVC epinephrine administration. With the recent suggestion to initiate epinephrine at 0.02 mg/kg/dose, we compared 0.02 mg/kg with 0.03 mg/kg/dose through both routes. Our primary outcome measures were the time to first epinephrine administration and the time to ROSC, and the secondary outcome measures were the incidence of ROSC and epinephrine pharmacokinetics.

## 2. Materials and Methods

### 2.1. Animal Preparation

The study protocol was approved by the Institutional Animal Care and Use Committee (IACUC; protocol #20734) at the University of California Davis, Davis, CA, USA. All experiments were performed according to animal ethical guidelines, in compliance with the ARRIVE guidelines [24], as previously described in [25]. Time-dated near-term (139–141-day gestation; term of 145 days) Dorper cross pregnant ewes were transported from Van Laningham Farm, Arbuckle, CA, USA. Following an overnight fast, the ewes were medicated with IV diazepam and ketamine. Subsequently, the ewes were intubated with a 9.5 or 10 mm cuffed ETT, and general anesthesia was provided by 2–3% inhaled isoflurane. The ewes were continuously monitored with a pulse oximeter and an end-tidal CO_2_ monitor. Following a cesarean section, the head and neck of the fetal lambs were partially exteriorized and intubated with a 4.5 mm cuffed ETT. The fetal lung fluid in the ETT was partially drained passively by gravity by tilting the head to the side. The ETT was subsequently occluded to prevent gas exchange during fetal gasping. A catheter was placed in the right carotid artery to measure blood pressure and collect blood samples. The right jugular vein was catheterized for fluid and medication administration. A left carotid ultrasound flow probe (3 mm) was placed to measure blood flow. A pulse oximeter was placed on the right forelimb for continuous oxygen saturation monitoring. Following instrumentation, the umbilical cord was occluded and cut at the placental end to leave a long (>20 cm) segment attached to the lamb. The lambs were immediately weighed after delivery to calculate the correct dose of epinephrine [10]. The laboratory had a large timer on display that was reset when cord occlusion was started for arrest and then reset again to zero when arrest occurred and, finally, when resuscitation was started. A research assistant exclusively watched and recorded the events and timing.

### 2.2. Experimental Protocol (Figure 1)

A five-minute period of asystole was observed prior to initiating resuscitation. Asystole was defined by the absence of carotid blood flow, arterial blood pressure, and HR. Resuscitation followed current NRP guidelines. Positive pressure ventilation (PPV) was provided by means of a T-piece resuscitator at pressures of 35/5 cm H_2_O at a rate of 40 breaths/min using 21% O_2_. Following 30 s of ventilation, chest compressions at a compression-to-ventilation ratio of 3:1 commenced with a simultaneous increase in inspired oxygen to 100%. Upon the initiation of chest compressions, the preparation to administer epinephrine began. The first dose of epinephrine was given as soon as intravenous access was established. Plasma epinephrine concentrations and arterial blood gases were collected at fetal baseline, asystole, one minute before and one minute after epinephrine administration, at the time of ROSC, and one minute, five minutes, ten minutes, and fifteen minutes after ROSC. Hemodynamic parameters were monitored continuously and recorded using data acquisition software. Plasma epinephrine concentrations were determined via liquid chromatography–mass spectrometry. 

**Figure 1:**
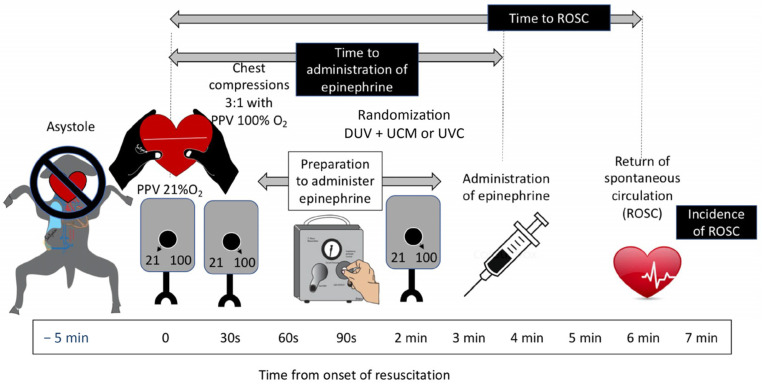
Resuscitation protocol: The umbilical cord was compressed to induce asystole in lambs. After 5 min of asystole, positive pressure ventilation (PPV) with a T-piece resuscitator with 21% oxygen was initiated. Thirty seconds later, inspired oxygen was increased to 100%, and chest compressions were initiated. At that time, preparation for epinephrine administration by direct injection or through a low umbilical vein catheter (per randomization allocation) began. Time to first epinephrine was calculated from the onset of PPV to administration of epinephrine.

### 2.3. Randomization

The lambs were randomized to one of four groups, either the intervention (DUV + UCM) or control (UVC EPI) at a dose of 0.02 or 0.03 mg/kg by drawing pre-labeled slips from an opaque envelope. Providers were not blinded to the epinephrine dose or route. Direct injection and catheter insertion were performed by either a neonatal fellow (PC) or attending physician (PV), providers with significant experience in umbilical vein catheter placement in clinical situations, animal handling, and procedures. The preparation for administering epinephrine was initiated after the onset of chest compressions.

**Figure 2:**
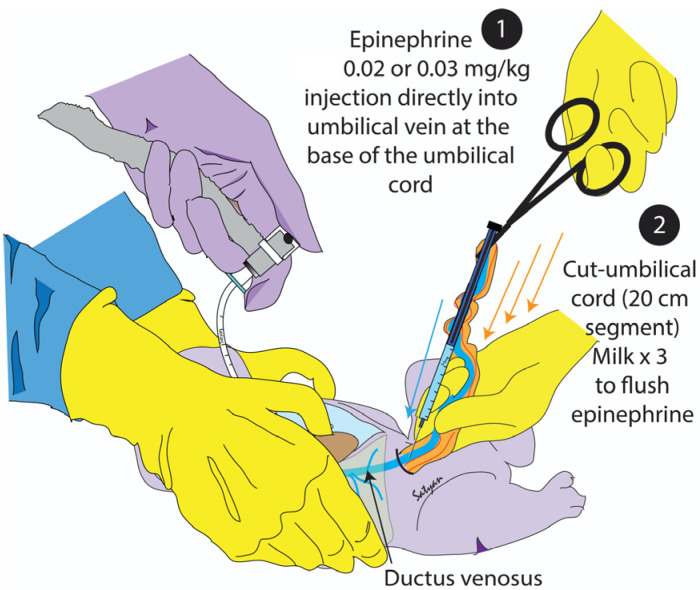
Schematic of DUV + UCM method: Experimental method of administering epinephrine by direct injection into the vein (blue arrow) at the base of long cut segment of the umbilical cord. The umbilical cord was milked to propel epinephrine into the neonate’s heart (orange arrows) using fetal blood in the cut segment of the umbilical cord.

*Intervention group (DUV + UCM):* The appropriate volume of epinephrine was drawn into a 1 mL 23-gauge (G) needle syringe and was inserted into the UV at the base of the umbilical cord at approximately a 30-degree angle. After confirming placement by checking for blood by drawing back the plunger, epinephrine was injected, and the needle was removed. The umbilical cord was milked in quick succession three times (Figure 2). If ROSC was not achieved, a low-UVC was placed, and the same dose of epinephrine was repeated every 3 min followed by a 3 mL normal saline flush until ROSC was achieved or after a total of 15 min of resuscitation. The lambs were monitored for one hour post-ROSC. 

*Control group (UVC EPI):* An umbilical venous catheter set was at the bedside. The UVC materials were not pre-connected or pre-flushed. The provider set up the catheter and stopcock and flushed it. Once a low-UVC was successfully placed, a syringe with the appropriate dose of epinephrine was administered through the catheter, followed by a 3 mL normal saline flush. If ROSC was not achieved, the same dose of epinephrine was repeated every 3 min until reaching ROSC. 

Resuscitation was continued until 15 min of resuscitation had elapsed. The lambs were monitored for one hour post-ROSC. 

### 2.4. Data Analysis

The sample size estimation was based on prior studies (including 31 lambs) with UVC epinephrine, where the mean time to ROSC was 4.5 min, with a standard deviation of 0.5 min. Assuming that the DUV + UCM group would achieve a 20% reduction in the ROSC time, we needed 4 lambs in each group to reject the null hypothesis with a power of 0.8 and type I probability of 0.05 [26]. Hemodynamic variables were continuously recorded at a sample rate of 2000 Hz using a computer with acquisition software (BIOPAC systems, version 5.0.7, Goleta, CA, USA). Continuous variables were expressed as means and the standard error of the mean (SEM). Categorical variables were analyzed using the χ^2^ test with Fisher’s exact test as required. The continuous variables were analyzed by two-way repeated measure ANOVA between groups, with Fisher’s post hoc test within groups. SPSS 24 (IBM, Armonk, NY, USA) was used for the statistical analysis. Statistical significance was defined as *p* < 0.05 [10]. 

## 3. Results

A total of 18 lambs were included in the analysis. The baseline characteristics are shown in Table 1. There was no significant difference between the groups, except that there were more female lambs in the DUV 0.02 mg/kg dose group, and this difference was marginally significant (*p* = 0.0503). The characteristics of asphyxia and the ROSC data are shown in Table 2. 

The time to asystole and the pH at asystole were similar between the four groups (Table 2). The partial pressure of oxygen, carbon dioxide, and lactate at asystole were similar between all four groups. 

### 3.1. Time to First Epinephrine, Number of Epinephrine Doses, and ROSC Efficacy

The overall incidence of ROSC was 16/18 (89%), with 9/10 of lambs in the DUV + UCM group achieving ROSC, compared with 7/8 in the UVC group. A total of 7 out of the 9 (78%) lambs in the DUV + UCM group achieved ROSC following the first dose of epinephrine, and 1 of the remaining 2 lambs was successfully resuscitated following epinephrine given through a UVC. Two out of the eight lambs in the UVC EPI group achieved ROSC prior to epinephrine administration. 

The mean time ± SEM to the first epinephrine dose from the onset of chest compressions was similar between the two groups (2.97 ± 0.48 min in the DUV + UCM group compared with 4.23 ± 0.58 in the control group; *p* = 0.12). The time to ROSC from the onset of PPV was similar between the groups (4.67 ± 0.67 min vs. 3.99 ± 0.58 min for DUV + UCM vs. UVC EPI, respectively; *p* = 0.58). Also, there was no difference in the time to ROSC from the first epinephrine administration (1.25 ± 0.20 min vs. 0.75 ± 0.17 min in the DUV + UCM and UVC EPI groups, respectively; *p* = 0.12). 

### 3.2. Plasma Epinephrine Pharmacokinetics (PK) after One Dose of Epinephrine

**Figure 3:**
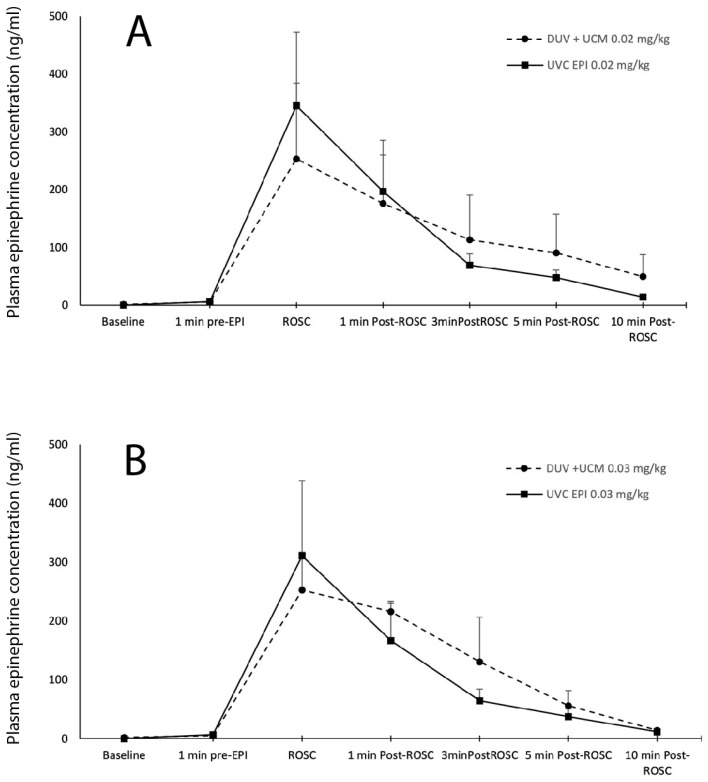
Plasma epinephrine concentration comparing DUV + UCM vs. UVC methods: there was no difference in plasma epinephrine concentrations between groups at (**A**) dose of 0.02 mg/kg and (**B**) dose of 0.03 mg/kg. DUV + UCM: direct umbilical vein followed by umbilical cord milking; UVC: umbilical vein catheter. Data are means ± SEM.

The plasma epinephrine concentrations between the two methods of administration were similar for each dose of 0.02 and 0.03 mg/kg throughout the study period (Figure 3A,B). The epinephrine plasma concentration peaked at the time of ROSC and exhibited a steady decline thereafter. The peak mean ± SEM epinephrine concentration in the DUV + UCM group at a dose of 0.02 mg/kg was 253 ± 131 ng/mL compared with 252 ± 65 ng/mL at 0.03 mg/kg. Following UVC EPI, the peak concentration at 0.02 mg/kg was 345 ± 127 ng/mL compared with 311 ± 142 ng/mL at 0.03 mg/kg. 

### 3.3. Hemodynamic Parameters

**Figure 4:**
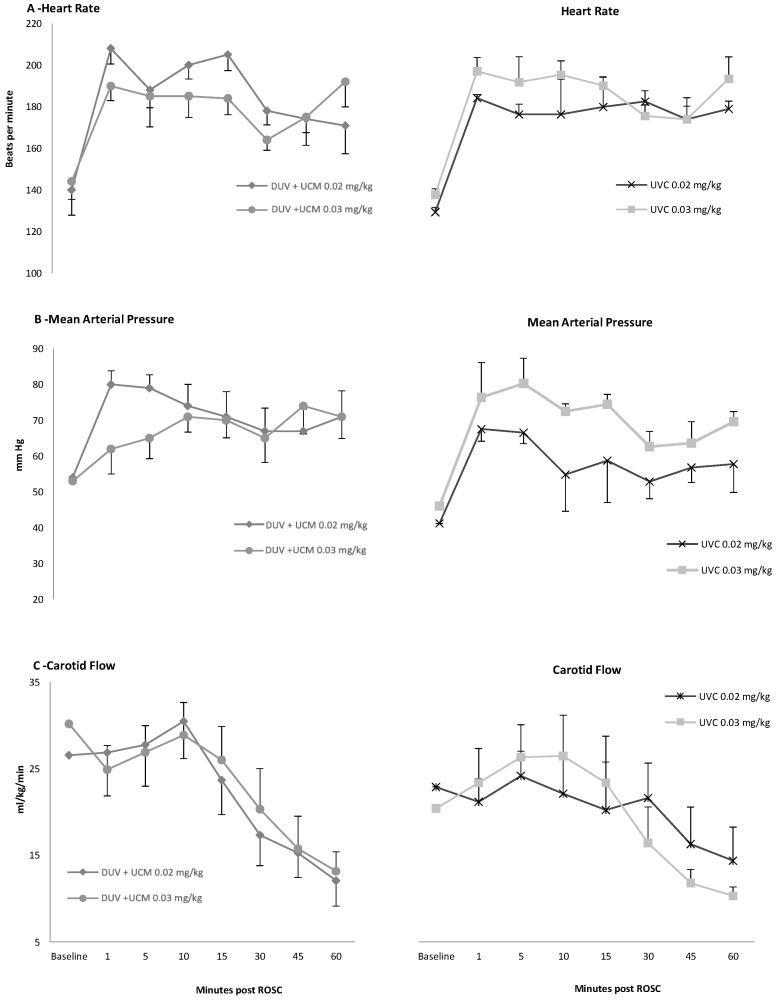
Hemodynamic parameters at baseline and post-return of spontaneous circulation: Heart rate significantly increased compared with baseline following ROSC and remained elevated with no difference between groups (**A**). Mean arterial blood pressure significantly increased compared with baseline with no differences between groups (**B**). Left mean carotid blood flow significantly decreased from baseline by 45 min following ROSC with no difference between groups (**C**). DUV + UCM: direct umbilical vein followed by umbilical cord milking; UVC: umbilical vein catheter. Data are means ± SEM.

The hemodynamic parameters at fetal baseline and in the one-hour period following ROSC are shown in Figure 4. There were no differences in heart rate, blood pressure, or left carotid blood flow between the two doses of epinephrine or between the two different administration methods at baseline and during the study period. The heart rate and mean arterial blood pressure increased immediately following the ROSC compared with the fetal baseline (Figure 4A,B). The mean left carotid blood flows reached fetal baseline values at 15 min post-ROSC (Figure 4C). 

## 4. Discussion

In this randomized trial, we demonstrated the feasibility of a direct umbilical vein injection followed by cut-cord milking to administer epinephrine during neonatal resuscitation. Nine out of ten lambs randomized to DUV + UCM achieved ROSC, and seven out of these nine lambs were successfully resuscitated following the first dose of epinephrine directly injected into the UV. Furthermore, our data show that plasma epinephrine concentrations are similar when epinephrine is directly injected into the base of the UV followed by cord milking compared with epinephrine injected into a UVC followed by a normal saline flush. 

Early administration of epinephrine during neonatal resuscitation is associated with a higher incidence of ROSC and may lead to better outcomes compared with delayed administration. In a recent retrospective study, 49% (561/1, 153) of neonates received epinephrine during cardiopulmonary resuscitation in the DR, and those who achieved ROSC had a significantly shorter time to administration of the first dose at 4 min (IQR 1, 8) compared with those who died in the DR and received the first dose at 7 min (IQR 1, 11) [5]. However, the time to the first epinephrine dose was not different between neonates who survived to discharge and those who died prior to discharge. The preparation of the UVC catheter, insertion, and epinephrine administration is a time-consuming process that requires both specific instruments and expertise. In this study, we demonstrated that direct injection of epinephrine into the UV followed by cut-cord milking is feasible. We speculate that the anatomical differences between species make it more difficult to inject the umbilical vein in lambs compared with humans (the ovine Wharton’s jelly is much more gelatinous, making it more difficult to anchor the umbilical vein).

The infrequency of epinephrine use in the DR has prevented the implementation of prospective clinical trials. Findings from some research on a perinatal asphyxiated lamb model with a transitioning circulation and fluid-filled lungs closely mimicking a newborn in the DR have recently informed NRP guidelines with respect to the flush volume following UVC administration of epinephrine, as well as a suggested optimal epinephrine dose [27,28]. We showed a 78% ROSC success rate with the DUV + UCM method, which was not statistically different from the administration of epinephrine through a UVC. Furthermore, for the two lambs that did not initially achieve the ROSC following DUV + UCM, one was successfully resuscitated after the placement of a UVC and a repeat epinephrine dose. These data suggest that epinephrine administration by DUV + UCM may be more effective than the endotracheal route, which had a 55% chance of ROSC success in a similar lamb model [10]. However, if there is no response to DUV + UCM, a UVC must be placed to administer epinephrine.

A recent ovine study did not show significant blood volume transfer in extremely preterm lambs that underwent umbilical cord milking without placental refill in a 10 cm cord segment [29]. However, previous work by our group has shown that a 2.5 mL flush is adequate to propel epinephrine from a low-UVC [30]. We hypothesize that the milking of the larger and longer (~20 cm) umbilical cord of near-term lambs transferred sufficient blood to successfully flush the medication into circulation. The bioavailability of epinephrine injection by DUV + UCM is similar to UVC-administered epinephrine as demonstrated by the similar pharmacokinetics between the groups (Figure 3). To our knowledge, this is the first published randomized study directly comparing the newly suggested dose of 0.02 mg/kg with 0.03 mg/kg. Our finding of an adequate plasma concentration and ROSC incidence support the current suggestion to initially administer a 0.02 mg/kg dose of epinephrine for IV (or IO) routes. 

Contrary to our hypothesis, DUV-administered epinephrine was not quicker compared with the placement of a UVC followed by an epinephrine injection. An alternate source for epinephrine administration is intraosseous administration by the insertion of an IO device. Pharmacokinetic studies have demonstrated equivalent drug plasma concentrations comparing IV with IO drug administration [31,32,33], and a recent study in neonatal lambs showed a similar bioavailability of epinephrine with IO and jugular vein administration [33]. IO device placement also requires specialized equipment and training [34]. An important advantage of the DUV + UCM method is that a 23–25 g needle syringe or butterfly is all that is required and can, therefore, be attempted in resource-limited and out-of-hospital environments. An additional syringe with flush is not required as the umbilical venous blood can propel the medication. In the lamb model, there was no bleeding from the puncture site with milking, and a UVC was successfully placed through the same site if a repeat dose of epinephrine was required.

We acknowledge several limitations to the current study. The small sample size along with species differences limit the clinical applicability of these findings. Ovine umbilical cord Wharton’s jelly is translucent, which allows easier identification of the umbilical vessels. However, the Wharton’s jelly is more gelatinous, which makes it more technically difficult to anchor the vein to directly inject epinephrine. The technique to insert a UVC in a lamb differs from how a UVC is inserted in a human newborn, and, therefore, the time of UVC placement cannot be compared between lambs and humans. Direct injection in the umbilical vein does pose an additional risk of needle-stick injury to neonatal providers. However, providers commonly draw umbilical arterial and venous gases with a similar technique in human neonates. We did not evaluate the brains of the lambs for evidence of injury. In cases of mild asphyxia, we can recover the lambs and follow neurodevelopmental changes [35]. However, with more profound arrest, we have not been able to resuscitate and extubate lambs and, hence, do not have data on the impact of DUV epinephrine on the brain. Finally, this study was not masked, and, hence, there is a possibility of bias from the provider during resuscitation.

## 5. Conclusions

In a perinatal asphyxial arrest near-term lamb model, epinephrine administration by DUV + UCM had a similar success of ROSC and bioavailability to UVC epinephrine. DUV + UCM may be considered as an alternate route of epinephrine administration in term or near-term infants while preparing for intravenous or intraosseus access. This technique does not require equipment such as a sterile umbilical venous catheter, sterile instruments, or a normal saline flush and, thus, can be used in resource-limited settings. Clinical studies are warranted to assess its feasibility in human neonates.

## Figures and Tables

**Table 1:** Baseline characteristics.

Group	DUV 0.02 mg/kg (*n* = 5)	UVC 0.02 mg/kg (*n* = 4)	DUV 0.03 mg/kg (*n* = 5)	UVC 0.03 mg/kg (*n* = 4)	*p*-Value
Weight (kg)	4.39 ± 0.15	4.17 ± 0.41	3.8 ± 0.37	3.69 ± 0.53	0.84
Sex (M:F)	0:5	2:2	4:1	3:1	0.05
Gestational age (days)	139 ± 0.2	141 ± 0.8	139 ± 0.4	139 ± 0.3	0.25
Baseline pH	7.19 ± 0.05	7.19 ± 0.02	7.20 ± 0.03	7.15 ± 0.05	0.59
Baseline PaCO_2_ (mm Hg)	69 ± 4.8	73 ± 4.7	75 ± 4.0	75 ± 6.4	0.60
Baseline PaO_2_ (mm Hg)	23 ± 3.3	17 ± 2.3	19 ± 3.1	15 ± 3.5	0.63
Baseline lactate (mmol/L)	2.8 ± 0.36	2.4 ± 0.48	4.2 ± 0.89	3.3 ± 0.94	0.72

Data are means ± SEM; DUV—direct umbilical vein injection; UVC—umbilical vein catheter.

**Table 2:** Asphyxia and return of spontaneous circulation (ROSC) data.

Group	DUV 0.02 mg/kg (*n* = 5)	UVC 0.02 mg/kg (*n* = 4)	DUV 0.03 mg/kg (*n* = 5)	UVC 0.03 mg/kg (*n* = 4)	*p*-Value
Time to asystole (min)	16.4 ± 1.2	14.6 ± 1.8	18.2 ± 1.6	14.4 ± 2.7	0.54
Asystole pH	6.91 ± 0.02	6.85 ± 0.02	6.90 ± 0.03	6.83 ± 0.15	0.48
Asystole PaCO_2_ (mm Hg)	122 ± 3.1	132 ± 8.4	133 ± 4.3	133 ± 2.6	0.24
Asystole PaO_2_ (mm Hg)	0.52 ± 0.35	1.4 ± 1.2	0.4 ± 0.2	0.3 ± 0.1	0.21
Asystole lactate (mmol/L)	7.1 ± 0.44	7.7 ± 0.44	8.1 ± 0.89	8.5 ± 0.53	0.87
ROSC	5/5 (100%)	3/4 (75%)	4/5 (80%)	4/4 (100%)	0.61
ROSC without EPI	0/5	1/4 (25%)	0/5	1/4 (25%)	1
Required > 1 EPI (if ROSC)	1/5 (20%)	0/3	1/4 (25%)	0/5	0.45
Time to 1st EPI (min)	2.24 ± 0.26	4.47 ± 0.92	3.7 ± 0.86	4.0 ± 0.89	0.11
Time to ROSC (minutes)	4.16 ± 0.89	3.66 ± 0.60	5.3 ± 0.9	4.24 ± 0.94	0.70
1st EPI to ROSC (min)	0.9 ± 0.26	0.71 ± 0.04	1.6 ± 0.38	0.77 ± 0.30	0.13

Data are means ± SEM. DUV: direct umbilical vein; UVC: umbilical vein catheter.

## Data Availability

The data presented in this study are available on request from the corresponding author. The data are not publicly available due to complex nature of the calculations and need for interpretation.

## References

[1] Hug L., Alexander M., You D., Alkema L., UN Inter-agency Group for Child Mortality Estimation (2019). National, regional, and global levels and trends in neonatal mortality between 1990 and 2017, with scenario-based projections to 2030: A systematic analysis. Lancet Glob. Health.

[2] Harrington D.J., Redman C.W., Moulden M., Greenwood C.E. (2007). The long-term outcome in surviving infants with apgar zero at 10 minutes: A systematic review of the literature and hospital-based cohort. Am. J. Obstet. Gynecol..

[3] Halling C., Sparks J.E., Christie L., Wyckoff M.H. (2017). Efficacy of intravenous and endotracheal epinephrine during neonatal cardiopulmonary resuscitation in the delivery room. J. Pediatr..

[4] Wyckoff M.H., Salhab W.A., Heyne R.J., Kendrick D.E., Stoll B.J., Laptook A.R., National Institute of Child Health and Human Development Neonatal Research Network (2012). Outcome of extremely low birth weight infants who received delivery room cardiopulmonary resuscitation. J. Pediatr..

[5] Halling C., Raymond T., Brown L.S., Ades A., Foglia E.E., Allen E., Wyckoff M.H., American Heart Association’s Get with the Guidelines–Resuscitation Investigators (2021). Neonatal delivery room cpr: An analysis of the get with the guidelines^®^-resuscitation registry. Resuscitation.

[6] Foglia E.E., Weiner G., de Almeida M.F.B., Wyllie J., Wyckoff M.H., Rabi Y., Guinsburg R., International Liaison Committee On Resuscitation Neonatal Life Support Task Force (2020). Duration of resuscitation at birth, mortality, and neurodevelopment: A systematic review. Pediatrics.

[7] Isayama T., Mildenhall L., Schmölzer G.M., Kim H.-S., Rabi Y., Ziegler C., Liley H.G. (2020). The route, dose, and interval of epinephrine for neonatal resuscitation: A systematic review. Pediatrics.

[8] Aziz K., Lee H.C., Escobedo M.B., Hoover A.V., Kamath-Rayne B.D., Kapadia V.S., Magid D.J., Niermeyer S., Schmölzer G.M., Szyld E. (2020). Part 5: Neonatal resuscitation: 2020 american heart association guidelines for cardiopulmonary resuscitation and emergency cardiovascular care. Circulation.

[9] Weiner G.M., Zaichkin J. (2021). Textbook of Neonatal Resuscitation (nrp).

[10] Vali P., Chandrasekharan P., Rawat M., Gugino S., Koenigsknecht C., Helman J., Jusko W.J., Mathew B., Berkelhamer S., Nair J. (2017). Evaluation of timing and route of epinephrine in a neonatal model of asphyxial arrest. J. Am. Heart Assoc..

[11] Wilkins R.G. (1985). Radial artery cannulation and ischaemic damage: A review. Anaesthesia.

[12] Rovamo L., Nurmi E., Mattila M.M., Suominen P., Silvennoinen M. (2015). Effect of a simulation-based workshop on multidisplinary teamwork of newborn emergencies: An intervention study. BMC Res. Notes.

[13] McKinsey S., Perlman J.M. (2016). Resuscitative interventions during simulated asystole deviate from the recommended timeline. Arch. Dis. Child. Fetal Neonatal Ed..

[14] Trevisanuto D., De Bernardo G., Res G., Sordino D., Doglioni N., Weiner G., Cavallin F. (2016). Time perception during neonatal resuscitation. J. Pediatr..

[15] Ersdal H.L., Singhal N. (2013). Resuscitation in resource-limited settings. Semin. Fetal Neonatal Med..

[16] Barber C.A., Wyckoff M.H. (2006). Use and efficacy of endotracheal versus intravenous epinephrine during neonatal cardiopulmonary resuscitation in the delivery room. Pediatrics.

[17] Vali P., Mathew B., Lakshminrusimha S. (2017). In quest of epinephrine’s optimal route and dose in neonatal cardiopulmonary resuscitation-are we there yet?. J. Pediatr..

[18] Halling C. (2017). Reply. J. Pediatr..

[19] Katheria A.C., Law B.H.Y., Poeltler D., Rich W., Ines F., Schmolzer G.M., Lakshminrusimha S. (2023). Cardiac and cerebral hemodynamics with umbilical cord milking compared with early cord clamping: A randomized cluster crossover trial. Early Hum. Dev..

[20] Katheria A.C., Clark E., Yoder B., Schmolzer G.M., Yan Law B.H., El-Naggar W., Rittenberg D., Sheth S., Mohamed M.A., Martin C. (2023). Umbilical cord milking in nonvigorous infants: A cluster-randomized crossover trial. Am. J. Obstet. Gynecol..

[21] Katheria A., Mercer J., Poeltler D., Morales A., Torres N., Lakshminrusimha S., Singh Y. (2023). Hemodynamic changes with umbilical cord milking in nonvigorous newborns: A randomized cluster cross-over trial. J. Pediatr..

[22] Bora R.L., Bandyopadhyay S., Saha B., Mukherjee S., Hazra A. (2023). Cut umbilical cord milking (c-ucm) as a mode of placental transfusion in non-vigorous preterm neonates: A randomized controlled trial. Eur. J. Pediatr..

[23] Hosono S., Mugishima H., Takahashi S., Takahashi S., Masaoka N., Yamamoto T., Tamura M. (2015). One-time umbilical cord milking after cord cutting has same effectiveness as multiple-time umbilical cord milking in infants born at <29 weeks of gestation: A retrospective study. J. Perinatol. Off. J. Calif. Perinat. Assoc..

[24] Kilkenny C., Browne W.J., Cuthill I.C., Emerson M., Altman D.G. (2010). Improving bioscience research reporting: The arrive guidelines for reporting animal research. PLoS Biol..

[25] Vali P., Chandrasekharan P., Rawat M., Gugino S., Koenigsknecht C., Helman J., Mathew B., Berkelhamer S., Nair J., Wyckoff M. (2017). Hemodynamics and gas exchange during chest compressions in neonatal resuscitation. PLoS ONE.

[26] Dupont W.D., Plummer W.D. (1998). Power and sample size calculations for studies involving linear regression. Control Clin. Trials.

[27] Vali P., Weiner G.M., Sankaran D., Lakshminrusimha S. (2021). What is the optimal initial dose of epinephrine during neonatal resuscitation in the delivery room?. J. Perinatol. Off. J. Calif. Perinat. Assoc..

[28] Sankaran D., Chandrasekharan P.K., Gugino S.F., Koenigsknecht C., Helman J., Nair J., Mathew B., Rawat M., Vali P., Nielsen L. (2021). Randomised trial of epinephrine dose and flush volume in term newborn lambs. Arch. Dis. Child. Fetal Neonatal Ed..

[29] Blank D.A., Polglase G.R., Kluckow M., Gill A.W., Crossley K.J., Moxham A., Rodgers K., Zahra V., Inocencio I., Stenning F. (2018). Haemodynamic effects of umbilical cord milking in premature sheep during the neonatal transition. Arch. Dis. Child. Fetal Neonatal Ed..

[30] Sankaran D., Vali P., Chandrasekharan P., Chen P., Gugino S.F., Koenigsknecht C., Helman J., Nair J., Mathew B., Rawat M. (2021). Effect of a larger flush volume on bioavailability and efficacy of umbilical venous epinephrine during neonatal resuscitation in ovine asphyxial arrest. Children.

[31] Brickman K.R., Rega P., Guinness M. (1987). A comparative study of intraosseous versus peripheral intravenous infusion of diazepam and phenobarbital in dogs. Ann. Emerg. Med..

[32] Von Hoff D.D., Kuhn J.G., Burris H.A., Miller L.J. (2008). Does intraosseous equal intravenous? A pharmacokinetic study. Am. J. Emerg. Med..

[33] Roberts C.T., Klink S., Schmolzer G.M., Blank D.A., Badurdeen S., Crossley K.J., Rodgers K., Zahra V., Moxham A., Roehr C.C. (2021). Comparison of intraosseous and intravenous epinephrine administration during resuscitation of asphyxiated newborn lambs. Arch. Dis. Childhood. Fetal Neonatal Ed..

[34] Rajani A.K., Chitkara R., Oehlert J., Halamek L.P. (2011). Comparison of umbilical venous and intraosseous access during simulated neonatal resuscitation. Pediatrics.

[35] Mike J.K., White Y., Hutchings R.S., Vento C., Ha J., Iranmahboub A., Manzoor H., Gunewardena A., Cheah C., Wang A. (2023). Effect of Clemastine on Neurophysiological Outcomes in an Ovine Model of Neonatal Hypoxic-Ischemic Encephalopathy. Children.

